# A neural mass model of spectral responses in electrophysiology

**DOI:** 10.1016/j.neuroimage.2007.05.032

**Published:** 2007-09-01

**Authors:** R.J. Moran, S.J. Kiebel, K.E. Stephan, R.B. Reilly, J. Daunizeau, K.J. Friston

**Affiliations:** a The Wellcome Trust Centre for Neuroimaging, Institute of Neurology, University College London, 12 Queen Square, London, WC1N 3BG, UK; b The School of Electrical, Electronic and Mechanical Engineering, University College Dublin, Dublin, Ireland

## Abstract

We present a neural mass model of steady-state membrane potentials measured with local field potentials or electroencephalography in the frequency domain. This model is an extended version of previous dynamic causal models for investigating event-related potentials in the time-domain. In this paper, we augment the previous formulation with parameters that mediate spike-rate adaptation and recurrent intrinsic inhibitory connections. We then use linear systems analysis to show how the model's spectral response changes with its neurophysiological parameters. We demonstrate that much of the interesting behaviour depends on the non-linearity which couples mean membrane potential to mean spiking rate. This non-linearity is analogous, at the population level, to the firing rate–input curves often used to characterize single-cell responses. This function depends on the model's gain and adaptation currents which, neurobiologically, are influenced by the activity of modulatory neurotransmitters. The key contribution of this paper is to show how neuromodulatory effects can be modelled by adding adaptation currents to a simple phenomenological model of EEG. Critically, we show that these effects are expressed in a systematic way in the spectral density of EEG recordings. Inversion of the model, given such non-invasive recordings, should allow one to quantify pharmacologically induced changes in adaptation currents. In short, this work establishes a forward or generative model of electrophysiological recordings for psychopharmacological studies.

## Introduction

Neural mass models of cortical neurons offer valuable insight into the generation of the electroencephalogram (EEG) and underlying local field potentials (LFPs), (Wendling et al., 2000; Lopes da Silva et al., 1976). One particular neural mass model, which is based on a biologically plausible parameterization of the dynamic behaviour of the layered neocortex, has been successfully used to generate signals akin to those observed experimentally, including small-signal harmonic components, such as the alpha band, and larger, transient, event-related potentials (Jansen and Rit, 1995). Our previous treatment of this model has focused largely on the time-domain, where numerical integration has been used to produce predictions of observed EEG responses (David et al., 2005) and to infer the posterior parameter densities using Bayesian inversion (David et al., 2006a,b, Kiebel et al., 2006). In this paper, we consider a treatment of an enhanced version of this model in the frequency domain.

The goal of neural mass modelling is to understand the neuronal architectures that generate electrophysiological data. This usually proceeds under quasi-stationarity assumptions. Key model parameters are sought, which explain observed changes in EEG spectra, particularly under pathological conditions (e.g., Liley and Bojak, 2005; Rowe et al., 2005). Recently, neural mass models have been used as forward or generative models of EEG and MEG data. Bayesian inversion of these models furnishes the conditional or posterior density of the physiological parameters. This means that real data can be used to address questions about functional architectures in the brain and pathophysiological changes that are framed in terms of physiological and synaptic mechanisms. In this work, we focus on electrophysiological measures in the spectral domain and ask which key parameters of the model determine the spectral response. When we refer to spectral response, we do not imply that there is some sensory or other event to which the dynamics are responding; we use response to denote the models response to endogenous stochastic input that is shaped by its transfer function. This analytic treatment is a prelude to subsequent papers, where we use the model described below as a probabilistic generative model to make inferences about neuromodulatory mechanisms using steady-state spectral responses.

There has been a considerable amount of work on neural field models of EEG in the spectral domain (Robinson, 2005, Robinson et al., 2001, 1997; Steyn-Ross et al., 1999; Wright and Liley, 1996). Here, we address the same issues using a neural mass model. Neural field models treat the cortex as a spatial continuum or field; conversely, neural mass models treat electrical generators as point sources (cf., equivalent current dipoles in forward models of EEG). We have used networks of these sources to model scalp EEG responses in the time-domain (David et al., 2006a,b; Kiebel et al., 2006; Garrido et al., 2007) and wanted to extend these models to the frequency-domain. The advantage of neural mass networks is that extrinsic coupling among the sources can be parameterized explicitly and estimated easily. Moreover, these networks can model remote cortical areas with different compositions. For example, cortical areas can differ considerably with regard to microcircuitry and the relative concentrations of neurotransmitter receptors. Structural anisotropy implicit in neural mass models can also be accommodated by neural field approaches: neural field models have been used to measure the effects of thalamocortical connectivity (extrinsic) anisotropies and have demonstrated their role in alpha splitting of EEG spectra (Robinson et al., 2003). However, a neural mass approach not only accommodates intrinsic differences in cortical areas but also enables laminar-specific cortico-cortical connections, which predominate in sensory systems (Felleman and Van Esses, 1991; Maunsell and Van Essen, 1983; Rockland and Pandya, 1979). This is particularly relevant for pharmacological investigations of, and interventions in, cognitive processing, where one needs to establish which changes are due to local, area-specific effects and which arise from long-range interactions (Stephan et al., 2006).

The advantage of frequency-domain formulations is that the spectral response is a compact and natural summary of steady-state responses. Furthermore, generative modelling of these responses allows one to assess the role of different parameters and specify appropriate priors. Finally, an explicit model of spectral responses eschews heuristics necessary to generate spectral predictions from the models time-domain responses. In what follows, we will assume that low-level parameters, such as active synaptic densities or neurotransmitter levels, remain constant under a particular experimental condition and mediate their influence when the cortical micro-circuit is at steady state.

This paper comprises three sections. In the first, we review the basic neural mass model, with a special focus on current extensions. In the second section, we take a linear systems perspective on the model and describe its various representations in state-space and the coefficients of its transfer function. In the third section, we use the transfer function to examine how steady-state spectral response changes with the models parameters.

## The neural mass model

The basic model, described in David and Friston (2003) has been extended here to include recurrent inhibitory–inhibitory interneuron connections and slow hyperpolarizing currents (e.g. mediated by slow calcium-dependent potassium channels; Faber and Sah, 2003) which are critical for spike-rate adaptation. In this report, we focus on the input–output behaviour of a single neuronal mass or source comprising three subpopulations. Transfer function algebra allows our characterization to be extended easily to a network of neuronal masses.

### The Jansen model

**Fig. 1:**
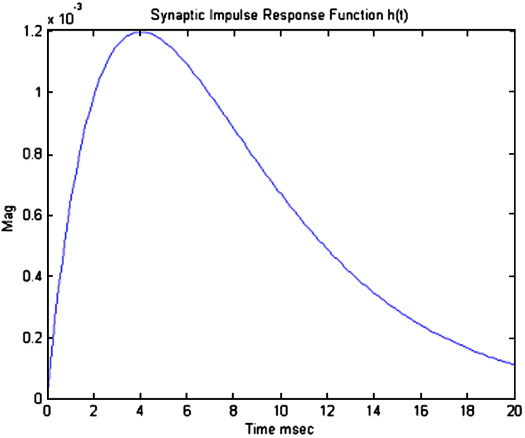
Synaptic impulse response function, convolved with firing rate to produce a postsynaptic membrane potential, parameter values are presented in Table 1.

A cortical source comprises several neuronal subpopulations. In this section, we describe the mathematical model of one source, which specifies the evolution of the dynamics of each subpopulation. This evolution rests on two operators: The first transforms *u*(*t*), the average density of presynaptic input arriving at the population, into *v*(*t*), the average postsynaptic membrane potential (PSP). This is modelled by convolving a parameterized impulse response function *h*_e/i_(*t*), illustrated in Fig. 1, with the arriving input. The synapse operates as either inhibitory or excitatory, (e/i), such that

(1) $$v(t)=h_{\text{e/i}}(t)⊗u(t)$$

$$h_{\text{e/i}}(t)=H_{\text{e/i}}\kappa_{\text{e/i}}t\exp\left(-t\kappa_{\text{e/i}}\right)$$

The parameter *H* tunes the maximum amplitude of PSPs and *κ* = 1/*τ* is a lumped representation of the sum of the rate constants of passive membrane and other spatially distributed delays in the dendritic tree. These synaptic junctions (in a population sense), allow a standard expansion from the kernel representation to a state-space formulation (David and Friston, 2003). The convolution leads to a second-order differential equation of the form

$$\overset{¨}{v}(t)=H_{\text{e/i}}\kappa_{\text{e/i}}u(t)-2\kappa_{\text{e/i}}\dot{v}(t)-\kappa_{\text{e/i}}^{2}v(t)$$

(Decomposed for state-space in normal form)

(2) $$\dot{v}(t)=x(t)$$

$$\dot{x}(t)=H_{\text{e/i}}\kappa_{\text{e/i}}u(t)-2\kappa_{\text{e/i}}x(t)-\kappa_{\text{e/i}}^{2}v(t)$$

The second operator transforms the average membrane potential of the population into the average rate of action potentials fired by the neurons. This transformation is assumed to be instantaneous and is described by the sigmoid function

(3) $$S(v)=\frac{1}{1+\exp\left(-\rho_{1}\left(v-\rho_{2}\right)\right)}-\frac{1}{1+\exp\left(\rho_{1}\rho_{2}\right)}$$

**Fig. 2:**
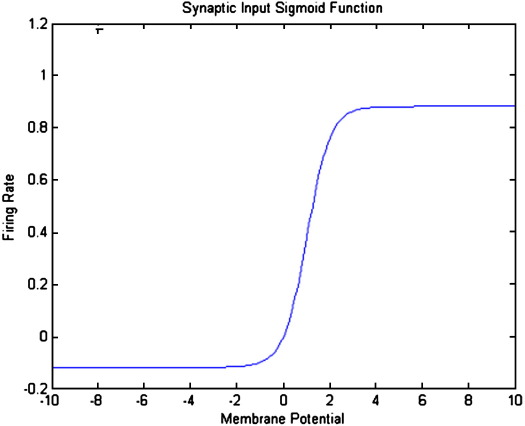
Non-linear sigmoid function for PSP to firing rate conversion, parameter values are presented in Table 1.

Where *ρ*_1_ and *ρ*_2_ are parameters that determine its shape (cf., voltage sensitivity) and position respectively, illustrated in Fig. 2. (Increasing *ρ*_1_ straightens the slope and increasing *ρ*_2_ shifts the curve to the right.) It is this function that endows the model with nonlinear behaviours that are critical for phenomena like phase-resetting of the M/EEG. Its motivation comes from the population or ensemble dynamics of many units and it embodies the dispersion of thresholds over units. Alternatively, it can be regarded as a firing rate–input curve for the ensemble average.

**Fig. 3:**
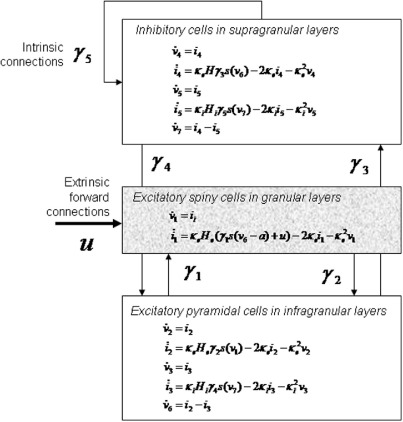
Source model, with layered architecture.

A single source comprises three subpopulations, each assigned to three cortical layers (Fig. 3).^1^ Following David et al., 2006a,b, we place an inhibitory subpopulation in the supragranular layer. This receives inputs from (i) the excitatory deep pyramidal [output] cells in an infra-granular layer, leading to excitatory postsynaptic potentials (EPSPs) *H*_e_ mediated by the coupling strength between pyramidal cells and inhibitory interneurons, *γ*_3_ and (ii) recurrent connections from the inhibitory population itself, which give rise to inhibitory postsynaptic potentials (IPSPs), *H*_i_ within the inhibitory population that are mediated by the inhibitory–inhibitory coupling parameter *γ*_5_. The pyramidal cells are driven by excitatory spiny [input] cells in the granular layer producing EPSPs mediated by coupling strength *γ*_2_, and inhibitory input from the interneurons providing IPSPs mediated through parameter *γ*_4_. Input to the macrocolumn, *u*, is through layer IV stellate cells, this population also receives excitatory (EPSPs) from pyramidal cells via *γ*_1_ (Fig. 3). Since the synaptic dynamics in Eq. (1) are linear, subpopulations are modelled with linearly separable synaptic responses to excitatory and inhibitory inputs. The state equations for these dynamics are

*Inhibitory cells in supragranular layers*
  $${\dot{v}}_{4}=i_{4}$$
  $${\dot{i}}_{4}=\kappa_{\text{e}}H_{\text{e}}\gamma_{3}S\left(v_{6}\right)-2\kappa_{\text{e}}i_{4}-\kappa_{\text{e}}^{2}v_{4}$$
  $${\dot{v}}_{5}=i_{5}$$
  $${\dot{i}}_{5}=\kappa_{\text{i}}H_{\text{i}}\gamma_{5}S\left(v_{7}\right)-2\kappa_{\text{i}}i_{5}-\kappa_{\text{i}}^{2}v_{5}$$
  $${\dot{v}}_{7}=i_{4}-i_{5}$$
*Excitatory spiny cells in granular layers*
  $${\dot{v}}_{1}=i_{1}$$
  $${\dot{i}}_{1}=\kappa_{\text{e}}H_{\text{e}}\left(\gamma_{1}S\left(v_{6}-a\right)+u\right)-2\kappa_{\text{e}}i_{1}-\kappa_{\text{e}}^{2}v_{1}$$
*Excitatory pyramidal cells in infragranular layers*
  (4) $${\dot{v}}_{2}=i_{2}$$
  $${\dot{i}}_{2}=\kappa_{\text{e}}H_{\text{e}}\gamma_{2}S\left(v_{1}\right)-2\kappa_{\text{e}}i_{2}-\kappa_{\text{e}}^{2}v_{2}$$
  $${\dot{v}}_{3}=i_{3}$$
  $${\dot{i}}_{3}=\kappa_{\text{i}}H_{\text{i}}\gamma_{4}S\left(v_{7}\right)-2\kappa_{\text{i}}i_{3}-\kappa_{\text{i}}^{2}v_{3}$$
  $${\dot{v}}_{6}=i_{2}-i_{3}$$
where *v*_i_ describe the membrane potentials of each subpopulation and *i*_i_, their currents. The pyramidal cell depolarization *v*_6_ = *v*_2_ − *v*_3_, represents the mixture of potentials induced by excitatory and inhibitory currents, respectively, and is the measured local field potential output.

These equations differ from our previous model (David et al., 2003) in three ways: first, the nonlinear firing rate–input curve in Eq. (3) has a richer parameterization, allowing for variations in its gain. Second, the inhibitory subpopulation has recurrent self-connections that induce the need for three further state variables (*i*_5_, *v*_5_, *v*_7_). Their inclusion into the model is motivated by experimental and theoretical evidence that such connections are necessary for high-frequency oscillations in the gamma band (40–70 Hz) (Vida et al., 2006; Traub et al., 1996; Wright et al., 2003).

Finally, this model includes spike-rate adaptation, which is an important mechanism mediating many slower neuronal dynamics associated with short-term plasticity and modulatory neurotransmitters (Faber and Sah, 2003; Benda and Herz, 2003).

#### Spike-frequency adaptation

The variable *a* in Eq. (4) represents adaptation; its dynamics conform to the universal phenomenological model described in Benda and Herz (2003, Eq. (3.1)).

(5) $$\dot{a}=\kappa_{a}\left(S\left(v\text{,}\;a\right)-a\right)$$

S(v,a)=S(v−a)

Adaptation alters the firing of neurons and accounts for physiological processes that occur during sustained activity. Spike-frequency adaptation is a prominent feature of neural dynamics. Various mechanisms can induce spike-rate adaptation (see Benda and Herz 2003 for review). Prominent examples include voltage-gated potassium currents (M-type currents), calcium-gated potassium channels (BK channels) that induce medium and slow after-hyperpolarization currents (Faber and Sah, 2003), and the slow recovery from inactivation of the fast sodium current (due to a use dependent removal of excitable sodium channels). The time constants of these processes range from a few 100 ms to 1–2 s.

Adaptation is formulated in Eq. (5) as a shift in the firing rate–input curve for two reasons; first, all three processes above can be reduced to a single *subtractive* adaptation current. Second, the time course of this adaptation is slow (see above), which means spike generation and adaptation are un-coupled. Eq. (5) represents a universal model for the firing-frequency dynamics of an adapting neuron that is independent of the specific adaptation and spike-generator processes.

**Fig. 4:**
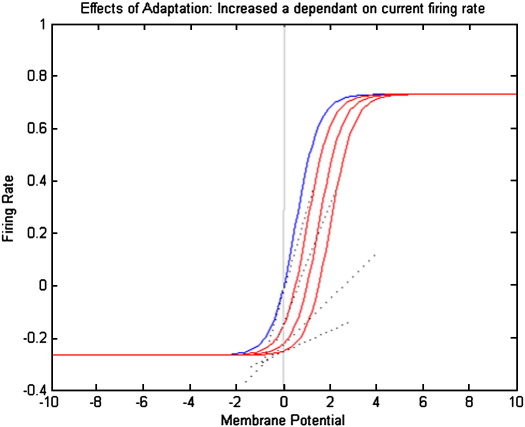
Phenomenological model of adaptation: increasing the value of *a* in Eq. (5) shifts the firing rate curve to the right and lowers its gain (as approximated linearly by a tangent to the curve at *v* = 0).

Adaptation depends on, and decays toward, the firing rate sigmoid function *S* at any given time: *S*(*v*,*a*) this is the steady state adaptation rate beyond which the neuron does not fire. The dynamics of adaptation is characterized by an intrinsic time constant *κ*_a_*.* Increasing *a* shifts the firing–input curve to the right (Fig. 4), so that a greater depolarization is required to maintain the same firing rate. Because this curve is nonlinear, the local gain or slope also changes. In the model here, only the spiny cells receiving extrinsic inputs show adaptation. This completes the description of the model and its physiological motivation. In the next section, we look at how this system can be represented and analysed in a linear setting.

## Linear representations of the model

Following the extension of the Jansen model to include the effects of recurrent inhibitory connections and adaptation currents, there are twelve states plus adaptation, whose dynamics can be summarized in state-space as

(6) $$\dot{x}=f(x)+Bu$$

y=Cx+Du

where *y* = *v*_6_ represents pyramidal cell depolarization, or observed electrophysiological output, and *x* includes all voltage and current variables, *v*_1–7_ and *i*_1–5_. The function *f*(*x*) is nonlinear due to the input–pulse sigmoid curve (Fig. 2). The form of *S*(*v*) means that the mean firing rate *S*(0) = 0 when the membrane potential is zero. This means the states have resting values *x*_0_ = 0 of zero. Input to the stellate cell population is assumed at baseline with *u* = *0*. A linear approximation treats the states as small perturbation about this expansion point, whose dynamics depends on the slope of *S*(0). We refer to this as the sigmoid ‘gain’ *g*:

(7) $$\frac{\partial S(0)}{\partial v}=g=\frac{\rho_{1}\exp\left(\rho_{1}\rho_{2}\right)}{{\left(1+\exp\left(\rho_{1}\rho_{2}\right)\right)}^{2}}$$

Increasing *ρ*_1_ will increase the gain *g* = ∂*S*/∂*v* in terms of firing as a function of depolarization.

In what follows we are only interested in steady-state responses. The influence of adaptation will be assessed by proxy, through its effect on the gain. Above we noted that increasing adaptation shifts the sigmoid function to the right. Hence it effectively decreases the functional gain, *g*, similar to a decrease in *ρ*_1_. This can be seen in Fig. 4 as a decreased slope of the tangent to the curve at *v* = 0 (dotted lines).

The biological and mean-field motivations for the state-equations have been discussed fully elsewhere (David and Friston, 2003; David et al., 2004, 2006). Here, we focus on identification of the system they entail. To take advantage of linear systems theory, the system is expanded around its equilibrium point *x*_0_. By design, the system has a stable fixed-point at *x*_0_ = 0 ⇒ *x˙* = 0 (assuming baseline input and adaptation is zero; *u* = *a* = 0). This fixed point is found in a family of such points by solving the state equations *x˙* = 0. Using a different stable point from the family would amount to an increase or decrease in the DC output of the neural mass and so does not affect the frequency spectra. We can now recast to the approximate linear system

(8) $$\dot{x}=Ax+Bu$$

y=Cx+Du

$$A=\left(\begin{array}{cccccccccccc} 0 & 0 & 0 & 1 & 0 & 0 & 0 & 0 & 0 & 0 & 0 & 0\\ 0 & 0 & 0 & 0 & 1 & 0 & 0 & 0 & 0 & 0 & 0 & 0\\ 0 & 0 & 0 & 0 & 0 & 1 & 0 & 0 & 0 & 0 & 0 & 0\\ -\kappa_{\text{e}}^{2} & 0 & 0 & -2\kappa_{\text{e}} & 0 & 0 & 0 & 0 & \kappa_{\text{e}}H_{\text{e}}\gamma_{1}g & 0 & 0 & 0\\ \kappa_{\text{e}}H_{\text{e}}\gamma_{2}g & -\kappa_{\text{e}}^{2} & 0 & 0 & -2\kappa_{\text{e}} & 0 & 0 & 0 & 0 & 0 & 0 & 0\\ 0 & 0 & -\kappa_{\text{i}}^{2} & 0 & 0 & -2\kappa_{\text{i}} & 0 & 0 & 0 & 0 & 0 & \kappa_{\text{i}}H_{\text{i}}\gamma_{4}g\\ 0 & 0 & 0 & 0 & 0 & 0 & 0 & 1 & 0 & 0 & 0 & 0\\ 0 & 0 & 0 & 0 & 0 & 0 & -\kappa_{\text{e}}^{2} & -2\kappa_{\text{e}} & \kappa_{\text{e}}H_{\text{e}}\gamma_{3}g & 0 & 0 & 0\\ 0 & 0 & 0 & 0 & 1 & -1 & 0 & 0 & 0 & 0 & 0 & 0\\ 0 & 0 & 0 & 0 & 0 & 0 & 0 & 0 & 0 & 0 & 1 & 0\\ 0 & 0 & 0 & 0 & 0 & 0 & 0 & 0 & 0 & -\kappa_{\text{i}}^{2} & -2\kappa_{\text{i}} & \kappa_{\text{i}}H_{\text{i}}\gamma_{5}g\\ 0 & 0 & 0 & 0 & 0 & 0 & 0 & 1 & 0 & 0 & -1 & 0 \end{array}\right)\;B=\left(\begin{array}{c} 0\\ 0\\ 0\\ \kappa_{\text{e}}H_{\text{e}}\\ 0\\ 0\\ 0\\ 0\\ 0\\ 0\\ 0\\ 0 \end{array}\right)\;C^{T}=\left(\begin{array}{c} 0\\ 0\\ 0\\ 0\\ 0\\ 0\\ 0\\ 0\\ 1\\ 0\\ 0\\ 0 \end{array}\right)$$

where *D* = 0. This linearization gives state matrices *A*, *B*, *C* and *D*, where *A* = ∂*f*(*x*_0_)/∂*x* is a linear coefficient matrix also known as the system transfer matrix or Jacobian. Eq.(8) covers twelve states with parameters, *θ* = *H*_e_,*H*_i_,*κ*_e_,*κ*_i_,*κ*_a_,*γ*_1_,*γ*_2_,*γ*_3_,*γ*_4_,*γ*_5_,*g*; representing synaptic parameters, intrinsic connection strengths, and gain, *g*(*ρ*_1_,*ρ*_2_). Both variables *ρ*_1_ and *ρ*_2_ parameterize the sensitivity of the neural population to input and *ρ*_2_ has the same effect as adaptation. Research using EEG time-series analysis (surrogate data testing) and data fitting have shown that linear approximations are valid for similar neural mass models (Stam et al., 1999; Rowe et al., 2004). Seizure states, and their generation, however, have been extricated analytically using non-linear neural mass model analysis (Rodrigues et al., 2006; Breakspear et al., 2006). In this context, large excitation potentials and the role of inhibitory reticular thalamic neurons preclude the use of the model described here; this is because many of the important pathophysiological phenomena are generated (after a bifurcation) explicitly by non-linearity. For example, spike and slow-waves may rest on trajectories in phase-space that lie in the convex and concave part of a sigmoid response function, respectively. Our model can be viewed as modelling small perturbations to spectral dynamics that do not engage nonlinear mechanisms. Thus, the system can be analyzed using standard procedures, assuming time invariance or stationarity.

### System transfer function

The general theory of linear systems can now be applied to the neural mass model. For an in-depth introduction, the reader is directed to the text of Oppenheim et al. (1999). The frequency response and stability of systems like Eq. (8) can be characterized completely by taking the Laplace transform of the state-space input–output relationship; *i.e.*, *Y*(*s*) = *H*(*s*)*U*(*s*). This relationship rests on the transfer function of the system, *H*(*s*), which is derived using the state matrices. This system transfer function, *H*(*s*) filters or shapes the frequency spectra of the input, *U*(*s*) to produce the observed spectral response, *Y*(*s*). The Laplace transform

(9) $$F(s)=L\left\{f(t)\right\}=\int_{0^{-}}^{\infty }e^{-st}f(t)dt$$

introduces the complex variable *s* = *α* + *iω* to system analysis (as distinct to capital “*S*”, which in preceding sections denoted the sigmoid firing rate), where the real part *α* says whether the amplitude of the output is increasing or decreasing with time. The imaginary part indicates a periodic response at frequency of *ω*/2*π*. This is seen by examining the inverse Laplace transform, computed along a line, parallel to the imaginary axis, with a constant real part.

(10) $$y(t)=L^{-1}\left\{Y(s)\right\}=\frac{1}{2\pi i}\int_{\alpha -i\infty }^{\alpha +i\infty }Y(s)e^{st}ds$$

This will consist of terms of the form *y*_0_*e*^*αt*^*e*^*jωt*^ that represent exponentially decaying oscillations at *ω* rad^− 1^. The Laplace transform of Eq.(8) gives

(11) sX(s)=AX(s)+BU(s)

Y(s)=CX(s)+DU(s)

⇒

$$X(s)={\left(sI-A\right)}^{-1}BU(s)$$

Y(s)=H(s)U(s)

$$H(s)=C{\left(sI-A\right)}^{-1}B+D$$

The transformation from Eq. (8) to Eq. (11) results from one of the most useful properties of this particular (Laplace) transform, in so far as conversion to the Laplace domain from the time-domain enables differentiation to be recast as a multiplication by the domain parameter ‘*s*’. The transfer function *H*(*s*) represents a normalized model of the system's input–output properties and embodies the steady-state behaviour of the system. One computational benefit of the transform lies in its multiplicative equivalence to convolution in the time-domain; it therefore reduces the complexity of the mathematical calculations required to analyze the system. In practice, one usually works with polynomial coefficients that specify the transfer function. These are the poles and zeros of the system.

### Poles, zeros and Lyapunov exponents

In general, transfer functions have a polynomial form,

(12) $$H(s)=\frac{\left(s-\varsigma_{1}\right)\left(s-\varsigma_{2}\right)\left(s-\varsigma_{3}\right)\ldots }{\left(s-\lambda_{1}\right)\left(s-\lambda_{2}\right)\left(s-\lambda_{3}\right)\ldots }$$

The roots of the numerator and denominator polynomials of *H*(*s*) summarize the characteristics of any LTI (linear time-invariant) system. The denominator is known as the characteristic polynomial, the roots of which known as the system's *poles*, the roots of the numerator are known as the system's *zeros*. The poles are solutions to the characteristic equation *sI* − *A* = 0 in Eq. (11); this means the poles *λ* are the Jacobian's eigenvalues (such that *λ*_*i*_ = *v*_*i*_^−^*Av*_*i*_, where {*v*_1_, *v*_2_, *v*_3_, …} are the eigenvectors of the Jacobian A, and *v*^−^ denotes their generalized inverse). In general non-linear settings, Lyapunov exponents, *e*^*λt*^, describe the stability of system trajectories; chaos arises when a large positive exponent exists. Usually, the Lyapunov exponents are the expected exponents, over non-trivial orbits or trajectories. In our setting, we can view the poles as complex Lyapunov exponents evaluated at the system's fixed point, where stability properties are prescribed by the real part, *α* of *s* = *α* + *iω*: for oscillatory dynamics not to diverge exponentially, the real part of the poles must be non-positive. The eigenvectors *v* satisfy; *Av* = *vλ* and *v*^−^ are their generalized inverse. We will use these eigenvectors below, when examining the structural stability of the model.

The poles *λ*_*i*_ and zeros *ς*_*i*_ represent complex variables that make the transfer function infinite and zero, respectively; at each pole the transfer function exhibits a singularity and goes to infinity as the denominator polynomial goes to zero. Poles and zeros are a useful representation of the system because they enable the frequency filtering to be evaluated for any stationary input. A plot of the transfer function in the *s*-plane provides an intuition for the frequency characteristics entailed; “cutting along” the *jω* axis at *α* = 0 gives the frequency response

(13) $$g_{h}(\omega )=|H\left(j\omega \right){|}^{2}$$

$$H\left(j\omega \right)=\frac{\left(j\omega -\varsigma_{1}\right)\left(j\omega -\varsigma_{2}\right)\left(j\omega -\varsigma_{3}\right)\ldots }{\left(j\omega -\lambda_{1}\right)\left(j\omega -\lambda_{2}\right)\left(j\omega -\lambda_{3}\right)\ldots }$$

**Fig. 5:**
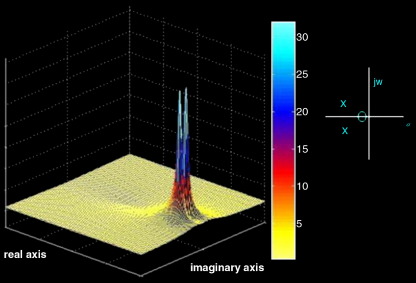
Example system’s frequency response on the complex *s*-plane, given two poles and one zero. The magnitude is represented by the height of the curve along the imaginary axis.

An example of a simple two-pole and one-zero system is illustrated in Fig. 5.

Eq. (13) can be used to evaluate the system's modulation transfer function, *g*_*h*_(*ω*), given the poles and zeros. We will use this in the next section to look at changes in spectral properties with the system parameters.^2^

The form of the transfer function encodes the dynamical repertoire available to a neuronal ensemble. The poles and zeros provide an insight into how parameters influence the system's spectral filtering properties. For example, the state-space characterization above tells us how many poles exist: This is determined by the Jacobian; since the transfer function is *H*(*s*) = *D* + *C*(*sI* − *A*)^− 1^*B*, the inverse part produces the transfer function's denominator. The order of the denominator polynomial is the rank of the Jacobian; here ten, this leaves two poles at the origin. The number of zeros is determined by all four system matrices. The sparse nature of these matrices leads to only two zeros. The transfer function for the neural model thus takes the form

(14) $$H(s)=\frac{\left(s-\varsigma_{1}\right)\left(s-\varsigma_{2}\right)}{s^{2}\left(s-\lambda_{1}\right)\ldots \left(s-\lambda_{10}\right)}$$

A partial analytic characterization of the poles and zeros shows that excitatory and inhibitory rate constants determine the locations of minimum and maximum frequency responses. Also one observes that inhibitory interneurons influence the location of the zeros. A complete analytic solution would allow a sensitivity analysis of all contributing parameters. Those coefficients that have been identified analytically include:

**Zeros:**
  (15) $$\varsigma_{1}=\frac{-\exp\left(\rho_{1}\rho_{2}\right)-1}{1+\exp\left(\rho_{1}\rho_{2}\right)\tau_{\text{i}}}+\frac{\sqrt{\rho_{1}\tau_{\text{i}}H_{\text{i}}\gamma_{5}\exp\left(\rho_{1}\rho_{2}\right)}}{1+\exp\left(\rho_{1}\rho_{2}\right)\tau_{\text{i}}}i$$
  $$\varsigma_{2}=\frac{-\exp\left(\rho_{1}\rho_{2}\right)-1}{1+\exp\left(\rho_{1}\rho_{2}\right)\tau_{\text{i}}}-\frac{\sqrt{\rho_{1}\tau_{\text{i}}H_{\text{i}}\gamma_{5}\exp\left(\rho_{1}\rho_{2}\right)}}{1+\exp\left(\rho_{1}\rho_{2}\right)\tau_{\text{i}}}i$$
**Poles:**
  (16) $$\lambda_{1}=-\kappa_{\text{e}}\;\lambda_{2}=-\kappa_{\text{e}}\;\lambda_{3}=-\kappa_{\text{i}}\;\lambda_{4}=-\kappa_{\text{i}}$$

All these poles are real negative. This indicates that associated eigenvectors or modes will decay exponentially to zero. Not surprisingly, the rate of this decay is proportional to the rate constants of synaptic dynamics. The remaining eigenvalues include four poles (two conjugate pairs) with complex eigenvalues that determine the system's characteristic frequency responses. These complex conjugate pairs result from a general property of polynomial equations; in so far as whenever the polynomial has real coefficients, only real and complex conjugate roots can exist. Unfortunately, there is no closed-form solution for these. However, this is not a problem because we can determine how the parameters change the poles by looking at the eigenvalues of the system's Jacobian. We will present this analysis in the next section. We can also compute which parameters affect the poles using the eigenvector solutions, *v*_*i*_^−^*Av*_*i*_ = *λ*_*i*_. This rests on evaluating

(17) $$\frac{\partial \lambda_{i}}{\partial \theta_{j}}=tr\frac{\partial \lambda_{i}}{\partial A}\frac{\partial A}{\partial \theta_{j}}=v_{i}^{-}\frac{\partial A}{\partial \theta_{j}}v_{i}$$

**Fig. 6:**
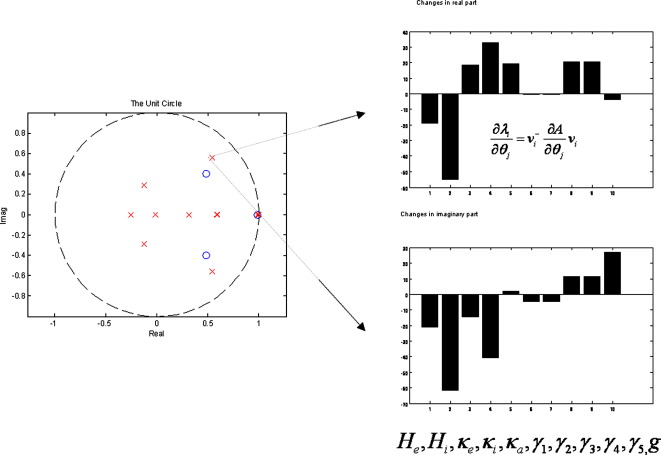
Sensitivity analysis: a pole depicted in the *z*-place (left) is characterized using eigenvector solutions (right) in terms of its sensitivity to changes in the parameters.

Here ∂*λ*_*i*_/∂*λ*_*j*_ encodes how the *i*-th pole changes with small perturbations of the *j*-th parameter (see Fig. 6 for an example).

Structural stability refers to how much the system changes with perturbations to the parameters. These changes are assessed in terms of stability in the face of perturbations of the system's states. The stable fixed point is lost if the parameters change too much. This is the basis of the zero-pole analysis that can be regarded as a phase-diagram. Eq. (4) can loose the stability of its fixed point, next, we look at how the Laplace transform can be used to establish the constraints on this system stability.

### Stability analysis in the *s*- and *z*-planes

In addition to providing a compact summary of frequency-domain properties, the Laplace transform enables one to see whether the system is stable. The location of the poles in the complex *s*-plane show whether the system is stable because, for oscillations to decay to zero, the poles must lie in the left-half plane (*i.e.*, have negative real parts). Conventionally, in linear systems analysis the poles of a digital system are not plotted in the *s*-plane but in the *z*-plane. The *z*-plane is used for digital systems analysis since information regarding small variations in sampling schemes can be observed on the plots (some warping of frequencies occurs in analog-to-digital frequency conversion). It is used here because this is the support necessary for the analysis of real data for which the scheme is intended; *i.e.* discretely sampled LFP time series.

The *z*-transform derives from the Laplace transform by replacing the integral in Eq. (9) with a summation. The variable *z* is defined as *z* = exp(*sT*), where *T* corresponds to the sampling interval. This function maps the (stable) left-half *s*-plane to the inside of the unit circle in the *z*-plane. The circumference of the unit circle is a (warped) mapping of the imaginary axis and outside the unit circle represents the (unstable) right-half *s*-plane. There are many conformal mappings from the complex plane to the *z*-plane for analogue to digital conversion. We used the bilinear transform since it is suitable for low sampling frequencies, where *z* = exp(*sT*) leads to the approximation

(18) $$s=\frac{2}{T}\frac{z-1}{z+1}$$

The *z*-transform is useful because it enables a stability analysis at a glance; critically stable poles must lie within the unit circle on the *z*-plane. We will use this in the analyses of the Jansen model described in the next section.

## Parameterized spectral output

In this section, we assess the spectral input–output properties of the model, and its stability, as a function of the parameters. In brief, we varied each parameter over a suitable range and computed the system's modulation transfer function, *g*_*h*_(*ω*) according to Eq. (13). These frequency domain responses can be regarded as the response to an input of stationary white noise; *g*_*y*_(*ω*) = *g*_*h*_(*ω*)*g*_*u*_(*ω*), when *g*_*u*_(*ω*) is a constant. To assess stability we tracked the trajectory of the poles, in the *z*-plane, as each parameter changed.

Each parameter was scaled between one quarter and four times its *prior* expectation. In fact, this scaling speaks to the underling parameterization of neural mass models. Because all the parameters *θ* are positive variables and rate constants, we re-parameterize with *θ*_*i*_ = *μ*_*i*_exp(*ϑ*_*i*_), where *μ*_*i*_ is the prior expectation (see Table 1) and *ϑ*_*i*_ is its log-scaling. This allows us to use Gaussian shrinkage priors on *ϑ*_*i*_ during model inversion. In this paper, we are concerned only with the phenomenology of the model and the domains of parameter space that are dynamically stable. However, we will still explore the space of *ϑ*_*i*_ because this automatically precludes inadmissible [negative] values of *θ*_*i*_.

The following simulations are intended to convey general trends in frequency response that result from changes in parameter values. Using standard parameter values employed in the SPM Neural Mass Model toolbox (http://www.fil.ion.ucl/spm), the system poles are stable and confined to the unit circle (Fig. 6). It can be seen that there are only two conjugate poles that have imaginary parts. These correspond to characteristic frequencies at about 16 Hz and 32 Hz.

**Fig. 7:**
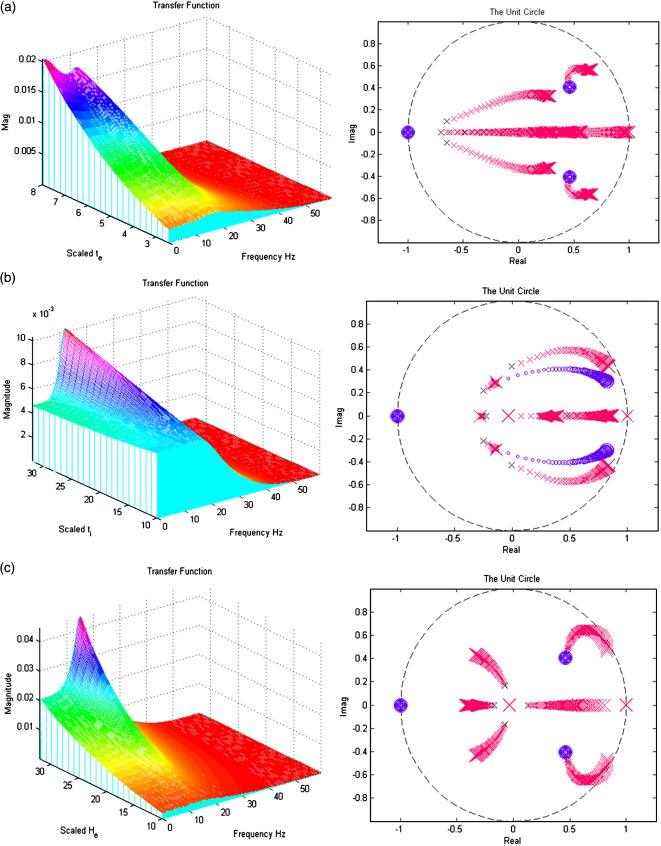

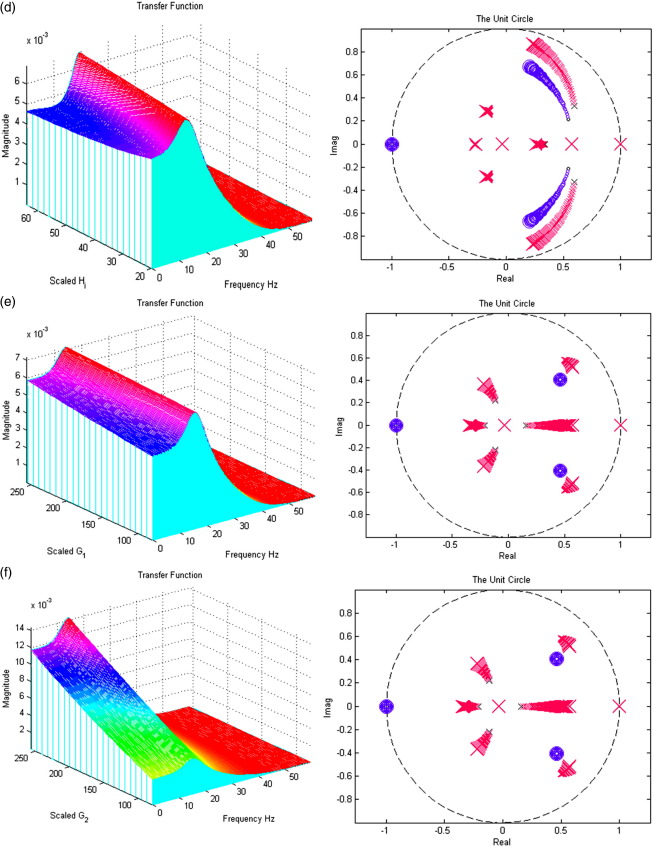

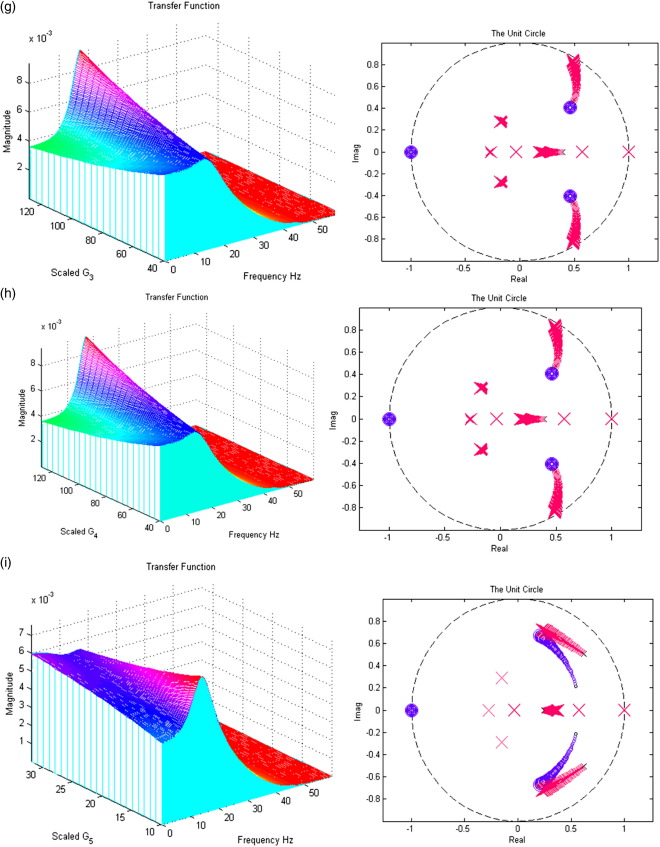
(a) The effect of variable excitatory time constant *τ*_e_. (b) The effect of variable inhibitory time constant *τ*_i_. (c) The effect of variable maximum excitatory postsynaptic potential *H*_e_. (d) The effect of variable maximum inhibitory postsynaptic potential *H*_i_. (e) The effect of variable pyramidal–pyramidal connection strength *γ*_1_. (f) The effect of variable stellate–pyramidal connection strength *γ*_2_. (g) The effect of variable pyramidal–inhibitory interneurons connection strength *γ*_3_. (h) The effect of variable inhibitory interneurons–pyramidal connection strength *γ*_4_. (i) The effect of variable inhibitory–inhibitory connection strength *γ*_5_.

In Figs. 7a–i; the left panels present a section of the modulation transfer functions, *g*_*h*_(*ω*) (viewed along the *x*-axis) as parameter scaling increases (moving back along the *y*-axis). The right figures present the movement of the poles and zeros to as the parameter increases from one quarter (small x/o) to four times (large X/O) its prior expectation (Figures may be viewed in colour on the web publication). For example, Fig. 7a shows how an increase in the excitatory lumped rate-constant, *τ*_e_ causes the poles nearest the unit circle to move even closer to it, increasing the magnitude of the transfer function. All parameters are set to prior values (Table 1) except that under test. Stability trends are disclosed with the accompanying *z*-plane plots.

Inspection of the poles and their sensitivity to changing the parameters is quite revealing. The first thing to note is that, for the majority of parameters, the system's stability and spectral behaviour are robust to changes in its parameters. There are two exceptions; the excitatory synaptic parameters (the time constant *τ*_e_ and maximum depolarization *H*_e_, in Figs. 7a and c, respectively). Increasing *τ*_e_ causes, as one might expect a slowing of the dynamics, with an excess of power at lower frequencies. Increasing *H*_e_ causes a marked increase and sharpening of the spectral mass of the lower-frequency mode. However, as with most of the parameters controlling the linear synaptic dynamics, there is no great change in the spectral composition of the steady-state response. This is in contrast to the effect of changing the gain (*i.e.*, sigmoid non-linearity).

**Fig. 8:**
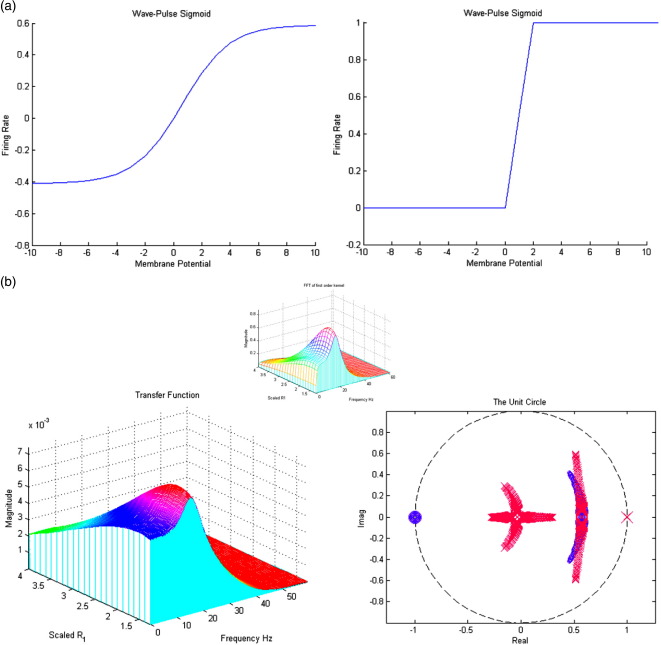
(a) Changing the gain through (left *ρ*_i_ = 1.25, right *ρ*_1_ = 4). (b) The effect of varying gain (inset: fft first-order non-analytic kernel using time-domain equation output).

This sigmoid function (Fig. 2) transforms the membrane potential of each subpopulation into firing rate, which is the input to other sources in network models. The efficacy of presynaptic ensemble input can be regarded as the steepness or gain of this sigmoid. We examined the effects of gain as a proxy for the underlying changes in the non-linearity (and adaptation). The transfer function plots in Fig. 8b correspond to making the sigmoid steeper (from the left to the right panels in Fig. 8a). There is a remarkable change in the spectral response as the gain is increased, with an increase in the characteristic frequency and its power. However, these effects are not monotonically related to the gain; when the gain becomes very large, stable band-pass properties are lost. A replication of the full system's time-domain response is shown in Fig. 8b (insert); comparing the modulation transfer function with the Fast Fourier Transform of the system's first-order (non-analytic) kernel, which represents the linear part of the entire output when a noise input is passed through the nonlinear system (see Friston et al., 2000).

In short, gain is clearly an important parameter for the spectral properties of a neural mass. We will pursue its implications in the Discussion.

In summary, the neural mass model is stable under changes in the parameters of the synaptic kernel and gain, showing steady-state dynamics, with a dominant high-alpha/low-beta rhythm. However, changes in gain have a more marked effect on the spectral properties of steady-state dynamics; increasing gain causes an increase in frequencies and a broadening of the power spectrum, of the sort associated with desynchronization or activation in the EEG. It should be noted that we have explored a somewhat limited parameter space in this paper. Profound increases in the recurrent inhibitory connections and gain can lead to much faster dynamics in the gamma range. Simulations (not shown) produced gamma frequency oscillations when the system was driven with very large inhibitory–inhibitory connection parameter, *λ*_5_. In this state, the pole placement revealed that the system was unstable. In this paper, however, our focus is on steady-state outputs elicited from ‘typical’ domains of parameter space, and does not elicit this gamma output.

## Discussion

We have presented a neural mass model for steady-state spectral responses as measured with local field potentials or electroencephalography. The model is an extended version of the model used for event-related potentials in the time-domain (Jansen and Rit, 1995; David et al., 2004, 2005, 2006a,b). We employed linear systems analysis to show how its spectral response depends on its neurophysiological parameters. Our aim was to find an accurate but parsimonious description of neuronal dynamics and their causes, with emphasis on the role of neuromodulatory perturbations. In this context, the model above will be used to parameterize the effects of drugs. This will enable different hypotheses about the site of action of pharmacological interventions to be tested with Bayesian model comparison, across different priors (Penny et al., 2004). Our model assumes stationarity at two levels. First, the data features generated by the model (*i.e.*, spectral power) are assumed to be stationary in time. Furthermore, the parameters of the model preclude time-dependent changes in dynamics or the spatial deployment of sources. This means we are restricted to modelling paradigms that have clear steady-state electrophysiological correlates (*e.g*., pharmacological modulation of resting EEG). However, there are some situations where local stationarity assumptions hold and may be amenable to modeling using the above techniques; for example, theta rhythms of up to 10 s have been recorded from intracranial human EEG during a visual working memory task (Radhavachari et al., 2001). Furthermore, there is no principal reason why the current model could not be inverted using spectra from a time–frequency analysis of induced responses, under the assumption of local stationarity over a few hundred milliseconds. This speaks to the interesting notion of using our linearized model in the context of a Bayesian smoother or updated scheme, to analyse the evolution of spectral density over time. The next paper on this model will deal with its Bayesian inversion using empirical EEG and LFP data.

Typical 1/*f* type EEG spectra are not as pronounced in our model as in many empirical spectra. The 1/*f* spectral profile, where the log of the spectral magnitude decreases linearly with the log of the frequency, is characteristic of steady-state EEG recordings in many cognitive states; and can be viewed as a result of damped wave propagation in a closed medium (Barlow, 1993; Jirsa and Haken, 1996; Robinson, 2005). This spectral profile has been shown to be insensitive to pre- and post-sensory excitation differences (Barrie et al., 1996), and represents non-specific desynchronization within the neural population (Lopez da Silva, 1991; Accardo et al., 1997). The parameters chosen to illustrate our model in this paper produce a well-defined frequency maximum in the beta range, which predominates over any 1/*f* profile. This reflects the fact that our model is of a single source and does not model damped field potentials, propagating over the cortex (*cf.*, Jirsa and Haken, 1996; Robinson, 2005). Second, we have deliberately chosen parameters that emphasize an activated [synchronized] EEG with prominent power in the higher [beta] frequencies to demonstrate the effects of non-linearities in our model. Adjusting the model parameters would place the maximum frequency in a different band (David et al., 2005); *e.g.*, the alpha band to model EEG dynamics in the resting state.

A potential issue here is that inverting the model with real data, displaying an underlying 1/*f* spectrum could lead to erroneous estimates of the parameters of interest, if the model tried to generate more low frequencies to fit the spectral data. However, this can be resolved by including a non-specific 1/*f* component as a confound, modelling non-specific components arising from distributed dynamics above and beyond the contribution of the source *per se*. Using Bayesian inversion, confounds like this can be parameterized and fitted in parallel with the parameters above, as fixed effects (Friston, 2002). Although the present paper is not concerned with model inversion, a forthcoming paper will introduce an inversion scheme which models the 1/*f* spectral component explicitly; this allows the model to focus on fitting deviations from the 1/*f* profile that are assumed to reflect the contribution of the source being modelled (Moran et al., submitted for publication). Furthermore, the inversion scheme allows priors on oscillations in particular frequency bands to be adjusted. This can be mediated through priors on specific parameters, e.g. *τ*_e/i_, that have been shown to modify resonant frequencies (David et al., 2005).

The present paper considers a single area but can be generalized to many areas, incorporating different narrow-band properties. Coupling among dynamics would allow a wide range of EEG spectra to be accommodated (David et al., 2003).

We have augmented a dynamic causal model that has been used previously to explain EEG data (David et al., 2006a,b) with spike-rate adaptation and recurrent intrinsic inhibitory connections. These additions increase the model's biological plausibility. Recurrent inhibitory connections have been previously investigated in spike-level models and have been shown to produce fast dynamics in the gamma band (32–64 Hz) (Traub et al., 1996). Similar conclusions were reached in the in vitro analysis of Vida et al. (2006), where populations of hippocampal rat neurons are investigated as an isolated circuit. In the present paper our focus was on the role of the sigmoid non-linearity (recast in a linear setting) linking population depolarization to spiking. We showed that much of the interesting spectral behaviour depends on this non-linearity, which depends on its gain and adaptation currents. This is important because, phenomenologically, adaptation currents depend on the activity of modulatory neurotransmitters: For example, the effects of dopamine on the slow Ca-dependent K-current and spike-frequency adaptation have been studied by whole-cell voltage-clamp and sharp microelectrode current-clamp recordings in rat CA1 pyramidal neurons in rat hippocampal slices (Pedarzani and Storm, 1995). Dopamine suppressed adaptation (after-hyperpolarization) currents in a dose-dependent manner and inhibited spike-frequency adaptation. These authors concluded that dopamine increases hippocampal neuron excitability, like other monoamine neurotransmitters, by suppressing after-hyperpolarization currents and spike-frequency adaptation, via cAMP and protein kinase A. Phenomenologically, this would be modelled by a change in *ρ*_2_, through implicit changes in gain associated with a shift of the sigmoid function to the left. The effect of dopamine on calcium-activated potassium channels is just one specific example (critically, these channels form the target for a range of modulatory neurotransmitters and have been implicated in the pathogenesis of many neurological and psychiatric disorders, see Faber and Sah, 2003 for review). Acetylcholine is known to also play a dominant role in controlling spike-frequency adaptation and oscillatory dynamics (Liljenstrom and Hasselmo, 1995), for example due to activation of muscarinergic receptors which induce a G-protein-mediated and cGMP-dependent reduction in the conductance of calcium-dependent potassium channels and thus prevent the elicitation of the slow after-hyperpolarization currents that underlie spike-frequency adaptation (Constanti and Sim, 1987; Krause and Pedarzani, 2006).

The key contribution of this paper is to show how activation of these slow potassium channels can be modelled by adding adaptation currents to a simple phenomenological model of EEG. Furthermore, using linear systems theory, we have shown that these changes are expressed in a systematic way in the spectral density of EEG recordings. Critically, inversion of the model, given such non-invasive recordings, should allow one to quantify pharmacologically induced changes in adaptation currents. This is the focus of our next paper (Moran et al., submitted for publication).

Limitations of this neural mass model include no formal mapping to conventional integrate-and-fire models in computational neuroscience. This is problematic because there is no explicit representation of currents that can be gated selectively by different receptor subtypes. On the other hand, the model partitions the linear and nonlinear effects neatly into (linear) synaptic responses to presynaptic firing and (nonlinear) firing responses to postsynaptic potentials. This furnishes a simple parameterization of the system's non-linearities and context-dependent responses. In summary, context-dependent responses are mediated largely though changes in the sigmoid non-linearity. Interestingly, these cover the effects of adaptation and changes in gain that have such a profound effect on steady-state dynamics. This is especially important for models of pharmacological intervention, particularly those involving classical neuromodulatory transmitter systems. These systems typically affect calcium-dependent mechanisms, such as adaptation and other non-linear (voltage-sensitive) cellular signalling processes implicated in gain.

## Figures and Tables

**Table 1:** Prior expectations of parameters

Parameter	Physiological interpretation	Standard prior mean
*H*_e/i_	Maximum post synaptic potentials	4 mV, 32 mV
*τ*_e/i_ = 1/*κ*_e/i_	Average dendritic and membrane rate constant	4 ms, 16 ms
*τ*_a_ = 1/*κ*_a_	Adaptation rate constant	512 ms
*γ*_1,2,3,4,5_	Average number of synaptic contacts among populations	128, 128, 64, 64, 16
*ρ*_1_, *ρ*_2_	Parameterized gain function *g*	2, 1

## Footnotes

1 Pyramidal cells and inhibitory interneurons are found in both infra- and supragranular layers in the cortex. However, note that the assignment of neuronal populations to layers is only important for multi-area models with connections that reflect the hierarchical position of the areas and thus have layer-specific terminations (see David et al., 2006a,b). The specific choice of which cell types should be represented in which layers does not make a difference as long as one models the cell-type-specific targets of forward, backward and lateral connections by appropriately constraining their origins and targets (compare Fig. 3 in David et al., 2005 with Fig. 3 in David et al., 2006a,b). Here, we have kept the assignment of cell types to layers as in David et al. (2006a,b).

2 This equality also highlights the close connection between the Laplace transform and the Fourier transform that obtains when *α* = 0.
